# Aging with burden: multimorbidity, depression and quality of life in older adults residing in long-term care facilities in South Africa

**DOI:** 10.1186/s12877-026-07846-4

**Published:** 2026-06-27

**Authors:** Shane Naidoo, Niri Naidoo

**Affiliations:** Division of Physiotherapy, Department of Health and Rehabilitation Sciences, University of Cape Town, Rondebosch, South Africa

**Keywords:** Older adults, Healthy aging, Depression, Multi-morbidity, Health-related quality of life, Physical activity

## Abstract

**Background:**

Population ageing has become an immediate global imperative, with older adults disproportionately afflicted by multimorbidity, a complex interplay of co-existing chronic diseases that erodes health-related quality of life (HRQoL). Multimorbidity is associated with impairments in physical and mental functioning, which undermine older adults’ autonomy and contributes to declines in HRQoL. However, vital data that explore these relationships remain absent for older populations residing in long-term care facilities (LTCFs) across sub-Saharan Africa. This study examines multimorbidity prevalence in LTCF residing older adults and its associations with depression, HRQoL outcomes and physical activity.

**Methods:**

A cross-sectional study among 396 older adults from 17 randomly selected South African LTCFs employed the Geriatric Depression Scale, EQ-5D-3 L, and Weighted Functional Comorbidity Index. Descriptive statistics, correlation analyses and TwoStep cluster analysis elucidated multimorbidity patterns.

**Results:**

Multimorbidity affected 76.77% of participants, with variation across four distinct clusters. The Cardiovascular-Respiratory cluster comprised 33.8% predominance. Multimorbidity was significantly predicted by poorer HRQoL and increasing age, χ²(140, *n* = 396) = 228.19, *p* < .001. Depression prevalence reached 35.10%, with higher scores associated with greater multimorbidity burden (ρ = 0.28, *p* < .01) and reduced HRQoL. An inverse correlation between the Weighted Functional Comorbidity Index and EQ-5D-3 L scores (ρ = −0.33, *p* < .001) reflected multimorbidity’s negative impact on HRQoL. Multimorbidity was inversely associated with Short Physical Performance Battery scores, lower physical activity, and directly to waist-to-hip ratios.

**Conclusion:**

Advancing age is strongly associated with an increased burden of multimorbidity, which is intricately linked to depressive symptoms, diminished HRQoL, reduced muscular strength, central adiposity, and physical inactivity. These findings highlight the urgent need for integrated interventions in institutionalised older adults in sub-Saharan Africa and should inform policy reform aimed at strengthening long-term care and healthy ageing strategies.

**Supplementary Information:**

The online version contains supplementary material available at https://doi.org/10.1186/s12877-026-07846-4.

## Background

As the population of older people increases globally, the cohort faces a mounting burden of chronic disease and mental health challenges [1–3]. Sub-Saharan Africa is progressively reflecting epidemiological patterns seen in other low- and middle-income countries, characterised by a rising prevalence of non-communicable diseases driven by demographic transitions, behavioural risk factors, and the compounded effects of environmental degradation and climate perturbations [2, 4]. Multi-morbidity is characterised by the concurrent presence of two or more chronic, long-term health conditions within a given person, with a higher prevalence observed in older adults [5, 6]. The 2016 South African Demographic and Health Survey reported multimorbidity rates of 47.2% in adults aged 60–69, 44.6% in those 70–79, and 43.7% among individuals aged 80+ [7].

Multimorbidity is strongly associated with a range of adverse outcomes, including diminished health-related quality of life (HRQoL), progressive functional impairment, increased disability, polypharmacy, and heightened healthcare expenditure, consequences that are especially pronounced in older populations [8–11]. These effects are compounded in countries where public health resources are already overstretched by the dual burden of infectious diseases and the high prevalence of trauma/violence-related injuries. Depression, as an independent comorbidity in older adults, is strongly associated with adverse clinical outcomes [12]. Furthermore, depression is frequently under-detected in older adults, despite being one of the most prevalent mental health disorders in later life, and a significant predictor of disability and mortality [13]. The odds of presenting with depression are significantly higher among older adults experiencing multimorbidity, with older women exhibiting a disproportionately greater risk [14, 15]. When co-occurring, multimorbidity and depression interact synergistically, establishing a reinforcing cycle that disproportionately accelerates the decline in HRQoL [14, 16].

Unlike many sub-Saharan African countries where institutional long-term care remains limited and family-based support is the dominant model, South Africa has a more established LTCF infrastructure, albeit insufficient for the population needs [17]. This reflects the country’s broader social protection system, urbanisation patterns, labour migration, and high burden of multimorbidity among older adults [17]. South African comparative research demonstrates that older adults living within community settings exhibit significantly higher physical activity levels and superior quality of life indices relative to residents of long-term care facilities, suggesting that ageing in place and sustained community integration may confer measurable functional and psychosocial advantages over institutional residence [18]. Although intergenerational care remains culturally valued, economic pressures and caregiver constraints increasingly necessitate institutional placement [19].

Despite emerging efforts to estimate multimorbidity prevalence in South Africa, existing evidence remains fragmented [20]. There remains a significant evidence gap regarding its prevalence and impacts on HRQoL and depression within LTCF in Sub-Saharan Africa. Rising demand for long-term care occupancy, driven by population ageing in this region, underscores the need for greater insight into these settings. The majority of LTCF in South Africa are privately and government funded/co-funded, with significant disparities in structural capacity and service delivery that are deeply rooted in historical inequities and shaped by socio-economic and geographic stratification [19, 21]. This study aims to investigate the prevalence of multimorbidity and its associations with HRQoL, depression and physical activity among older adults residing in South African LTCFs. Clarifying these interrelationships is vital to advancing person-centred care, optimising resource allocation, and shaping evidence-informed policies responsive to the needs of ageing societies.

## Methodology

### Design and setting

This study employed a cross-sectional design. Data collection was conducted across LTCFs located in both rural and urban areas within the eThekwini district of KwaZulu-Natal, South Africa. The eThekwini Municipality is South Africa’s third most populous municipality, with a population of 3.9 million and the fifth largest proportion (4.80%) of older adults among metropolitan areas [22]. The municipality comprises 44 registered LTCF housing 3,107 residents and is characterised by marked socio-cultural diversity, with 68% of its area classified as rural and nearly one-quarter of its population residing in informal settlements [23].

### Participants characteristics

Participants were male or female residents of LTCF within the eThekwini Municipality, aged ≥ 60 years, who passed the World Health Organization’s (WHO) Integrated Care for Older People (ICOPE) cognitive screening test and were able to provide informed consent. Exclusion criteria included inability to comprehend testing instructions, concurrent participation in other studies, use of medication affecting cognition or performance, treatment for or recent (< 7 days) recovery from acute illness, resting heart rate > 100 beats per minute, oxygen saturation < 96%, or resting blood pressure ≥ 139/99 mmHg.

### Recruitment

The sample size was calculated as *n* = 384 to achieve a 95% confidence interval, with recruitment increased to 400 to accommodate potential attrition. Reflecting the population distribution of the eThekwini Municipality, where 68% reside in urban areas and 32% in rural areas, stratified sampling was applied [22]. This approach resulted in the selection of seven urban and three rural LTCFs. From each of the ten LTCFs, 40 participants were randomly selected, provided they met the inclusion criteria. Stratified sampling was initially applied to select LTCF based on the urban–rural population distribution within the eThekwini Municipality, with an initial target of seven urban and three rural facilities. However, due to variability in resident eligibility and consent rates, the planned sample size could not be achieved from these facilities alone. Consequently, additional facilities were recruited sequentially until the required sample size for a 95% confidence interval was attained. In total, 17 LTCFs participated in the study. The recruitment of participants was undertaken between 18 and 29 September 2023, with subsequent screening procedures systematically administered in October 2023.

### Ethical considerations

Ethical clearance for the study was granted by the, University of Cape Town, Faculty of Health Sciences Human Research Ethics Committee (Ref: HREC/325/2023). Written informed consent was obtained from all participants, the respective randomly selected LTCF, the eThekwini Municipality, and the KwaZulu-Natal Department of Social Development. All ethical procedures, encompassing voluntary participation, confidentiality, and the right to withdraw without consequence, were rigorously upheld in accordance with established ethical standards. A clinical trial number was not necessary for this study.

### Outcome measurements

Intrinsic capacity was tested using the ICOPE screening tool, which assesses key domains of physical and mental capacity [24]. As part of the eligibility criteria, participants were required to pass the cognitive and hearing tests in the ICOPE screening, to ensure reliable self-reporting and valid engagement with the study procedures.

Following eligibility confirmation, a comprehensive battery of validated assessments was conducted to evaluate demographic, physiological, functional, and psychosocial outcomes:

- Sociodemographic characteristics included chronological age, biological sex, marital status, and educational attainment, were captured through a structured interview form.
- Anthropometric measurements were obtained using standardised procedures: weight and height were measured to calculate Body Mass Index (BMI) measured in kg/m^2^, while waist and hip circumferences were used to derive waist-to-hip ratio. According to WHO guidelines, the assessment of overweight and obesity should incorporate at least two anthropometric indicators, such as BMI and waist-to-hip ratio, to enhance diagnostic accuracy and account for variations in fat distribution [25].
- Physical function was assessed using:
  - ◦ A digital handgrip dynamometer to record handgrip strength to measure upper limb muscle strength. The top three readings per hand were recorded in kilograms (kg), with ≥ 15 s rest between trials to mitigate fatigue. Handgrip strength is a clinically validated biomarker of global muscular function and a strong prognostic indicator of frailty, morbidity, and adverse health outcomes in older adults [26].
  - ◦ The Short Physical Performance Battery (SPPB), which evaluates lower-extremity function through gait speed, static balance, and chair stand performance. Each component is scored on a scale of 0 to 4, with a cumulative total score ranging from 0 to 12 [27]. The SPPB has been utilised as a diagnostic tool for sarcopenia and is further recognised as a valid and reliable measure for identifying frailty among older adults in Sub-Saharan Africa [28, 29].
- Physical activity levels were measured using the International Physical Activity Questionnaire-Short Form, reporting energy expenditure in Metabolic Equivalent of Task (MET) minutes/week across various activity domains. According to MET scores, physical activity was delineated as low (< 600 MET minutes/week), moderate (≥600 and <3000 MET minutes/week), and high (≥ 3000 MET minutes/week). This is a validated and reliable instrument for assessing physical activity levels in older adults within the South African context, offering the advantage of brief administration time [30]. The number of sedentary minutes, as recorded in the questionnaire, was also included as an outcome measure to quantify sedentary behaviour among participants.
- Barriers to physical activity were assessed via the Barriers to Being Active Quiz, which explores psychological, social, and environmental constraints to regular physical activity [31, 32].
- Comorbidity burden was quantified using the weighted Functional Comorbidity Index (w-FCI), which assigns differential weights to chronic conditions based on their impact on physical functioning [33]. The w-FCI is a user-friendly, time-efficient instrument encompassing 18 comorbid conditions. It quantifies the severity of each condition in relation to an individual’s functional capacity and can be completed in under 10 min [33]. The w-FCI was utilised in this study for the following reasons:
  - ◦ It evaluates functional and physical impairment, in contrast to the Charlson Comorbidity Index, which primarily predicts mortality [34]. This distinction is critical as it enables the tailoring of physical activity interventions based on the prevalence and functional severity of comorbid conditions;
  - ◦ It provides a quantitative measure of the impact of comorbidities on functional performance in older adults [33].
  - ◦ For the purposes of this study, multimorbidity was operationally defined as the presence of two or more comorbid conditions identified within the w-FCI, consistent with established multimorbidity thresholds.
- Health-related quality of life was measured using the European Quality of Life 5 Dimensions 3 Level Version (EQ-5D-3L) instrument, with index scores derived using the Zimbabwean value set, reflecting regional contextual relevance. It is a self-reported, generic instrument designed to evaluate HRQoL and health status, irrespective of disease presence [35]. It has undergone validation for use within the South African context and is recognised for its brevity and ease of administration [36]. The tool is available in multiple official South African languages and has been validated for both contextualised research applications and routine clinical practice to assess HRQoL [37, 38].
- The Geriatric Depression Scale-Short Form (GDS) was utilised in this study to screen for depression. It consists of 15 items designed to screen for depressive symptoms in older populations. Scores are interpreted as follows: 0–4 indicates no depression, 5–8 suggests mild depression, 9–11 reflects moderate depression, and 12–15 denotes severe depression [39]. It was utilised for the following reasons:
  - ◦ A systematic review identified it as the most widely employed instrument for screening depression in older adults [39];
  - ◦ It has been previously applied in geriatric research within the South African context [40, 41];
  - ◦ It is considered appropriate for use among older adults residing in LTCF [39];
  - ◦ It is suitable for use in older adults with mild cognitive impairment, fatigue, or physical illness [42].
  - ◦ It is available in the isiZulu language, is freely accessible, and approved for use in academic research [43].
- Physiological measures included:
  - ◦ Resting heart rate systolic and diastolic blood pressure, assessed using an automated digital sphygmomanometer following standardised protocols and recorded in beats/minute and mmHg respectively.
  - ◦ Resting peripheral oxygen saturation (SpO₂), measured with a digital fingertip pulse oximeter after a minimum of two minutes at rest and recorded as a percentage.

## Statistical analysis

Statistical analysis utilising SPSS Version 27 was used to analyse data collected. Tests for normality were undertaken and descriptive statistics used. Data that were normally distributed were analysed using *t*-tests to check for differences between groups. The one-way ANOVA test was used to test the relationship between normally distributed ratio/continuous data and multiple level categorical variables. Data that was not distributed normally was analysed with the Mann-Whitney U test (Wilcoxon Rank-Sum test). The Kruskal-Wallis H test was used to test the relationship between ratio/continuous data that was not normally distributed and multiple level categorical data. Chi-squared analyses were used to check for differences in categorical data. Pearson’s correlation tests were used to analyse relationships between parametric data, with Spearman’s correlation tests used for non-parametric data. A TwoStep cluster analysis using SPSS Version 30 was utilised to identify distinct multimorbidity profiles within the study population. The analysis included categorical variables indicating the presence or absence of specific chronic conditions. The log-likelihood distance measure was employed to assess similarity between cases. The optimal number of clusters was determined based on model-fit criteria and interpretability of the cluster solutions. Identified clusters were subsequently characterised in terms of their key chronic disease patterns.

## Results

This section presents the principal findings of the study, focusing on the relationships between comorbidity burden, depressive symptomatology, and HRQoL among older adults residing in LTCFs.

### Sociodemographic characteristics, screening, and vital signs

In total 400 participants with informed consent were screened. Four participants were excluded from the study as per the eligibility criteria. One did not meet the criteria of the ICOPE screening tool due to cognitive impairment, while three presented with elevated blood pressure readings. They were subsequently referred to the LTCF resident nurse with an accompanying referral letter. Consequently, 396 participants were adjudged eligible for inclusion in the study. Outcome data on participant characteristics, clinical measures, and functional assessments are presented in Supplementary File 1.

#### Intrinsic screening

The depression component of the ICOPE screening tool demonstrated significant associations with GDS scores. Specifically, feelings of being down or depressed were significantly related to GDS scores (Wald χ² = 27.00, *df* = 14, *p* = .01), while reporting little interest in activities exhibited an even stronger association (Wald χ² = 48.37, *df* = 14, *p* < .001). These findings could support the potential screening efficacy of the ICOPE tool, suggesting its validity for identifying depressive symptoms in this population.

#### European quality of life 5 dimensions 3 level version

Pain emerged as the most frequently reported factor impacting HRQoL, cited by 46.6% of urban and 52.9% of rural participants (Table 1). Mobility limitations constituted the second most prevalent concern, followed by anxiety, as principal determinants adversely affecting participants’ HRQoL.

**Table 1:** European Quality of Life 5 Dimensions 3 Level Version (EQ-5D-3L) health-related quality of life (HRQoL)

EQ-5D-3L		Urban		Rural	
		Females (%)	Males (%)	Females (%)	Males (%)
Mobility	**No problems**	140 (66.67)	71 (71.72)	35 (76.09)	30 (73.17)
	**Some problems**	70 (33.33)	28 (28.28)	11 (23.91)	10 (24.39)
	**Extreme problems**	0	0	0	1 (2.44)
Self-care	**No problems**	198 (94.29)	94 (94.95)	44 (95.65)	40 (97.56)
	**Some problems**	12 (5.71)	5 (5.05)	2 (4.35)	1 (2.44)
	**Extreme problems**	0	0	0	0
Usual activities	**No problems**	177 (84.29)	87 (87.88)	43 (93.48)	37 (90.24)
	**Some problems**	31 (14.76)	12 (12.12)	3 (6.52)	4 (9.76)
	**Extreme problems**	2 (0.95)	0	0	0
Pain	**No problems**	111 (52.86)	54 (54.55)	18 (39.13)	23 (56.10)
	**Some problems**	77 (36.67)	43 (43.43)	24 (52.17)	16 (39.02)
	**Extreme problems**	22 (10.48)	2 (2.02)	4 (8.70)	2 (4.88)
Anxiety/ depression	**No problems**	145 (69.05)	65 (65.66)	35 (76.09)	36 (87.80)
	**Some problems**	50 (23.81)	31 (31.31)	10 (21.74)	5 (12.20)
	**Extreme problems**	15 (7.14)	3 (3.03)	1 (2.17)	0

Total Sample *n* = 396, Urban Female *n* = 210, Rural Female *n* = 46, Urban Male *n* = 99, Rural Male *n* = 41

**Fig. 1:**
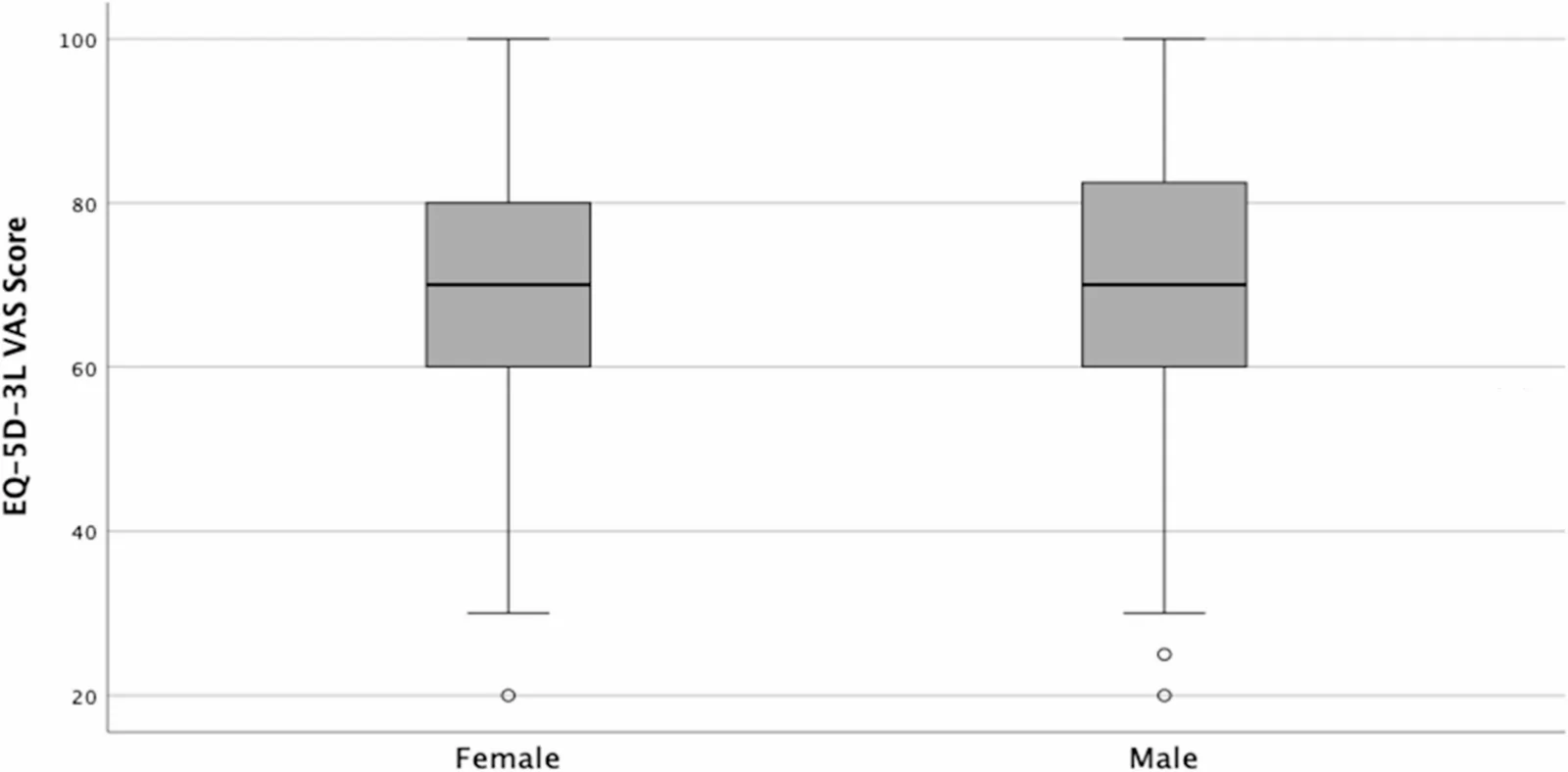
EQ-5D-3L visual analogue scale (VAS) score distribution

The mean VAS and index scores for the sample was 70.46 (95% CI [68.78, 72.14], SD 17.01) and 0.83 (95% CI [0.82, 0.84], SD 0.14) respectively. Males recorded higher VAS scores than females (Fig. 1).

Given the non-normal distribution of EQ-5D-3L index scores for both males and females, a Mann-Whitney U test indicated a statistically significant difference in index scores between males and females (*U* = 15655.00, *Z* = − 2.11, *p* = .04). The median values and interquartile ranges for EQ-5D-3L index scores are detailed in Table 2. There was a statistically significant negative correlation between GDS and EQ-5D-3L index scores, Spearman rho (*ρ*) = − 0.21, *p* < .001.

**Table 2:** European Quality of Life 5 Dimensions 3 Level Version (EQ-5D-3L) index scores

Age category (*n*)	Urban median index & IQR		Rural median index & IQR	
	Females	Males	Females	Males
60–65 yrs (75)	0.83 (0.77 − 1)	0.84 (0.77 − 1)	0.72 (0.59-0.83)	0.84 (0.83 − 1)
66–70 yrs (87)	0.83 (0.73 − 1)	0.83 (0.79-0.85)	1 (0.83 − 1)	0.83 (0.79-0.85)
71–75 yrs (91)	0.83 (0.73-0.86)	0.85 (0.83 − 1)	0.84 (0.83 − 1)	0.83 (0.69-0.92)
76–80 yrs (77)	0.83 (0.73-0.84)	0.81 (0.75-0.93)	0.81 (0.75-0.84)	0.84 (0.84-0.93)
81 yrs + (66)	0.83 (0.77 − 1)	0.83 (0.78 − 1)	0.83 (0.73 − 1)	0.86 (0.81 − 1)

Total Sample *n* = 396, Urban Female *n* = 210, Rural Female *n* = 46, Urban Male *n* = 99, Rural Male *n* = 41

*IQR* Interquartile range

**Fig. 2:**
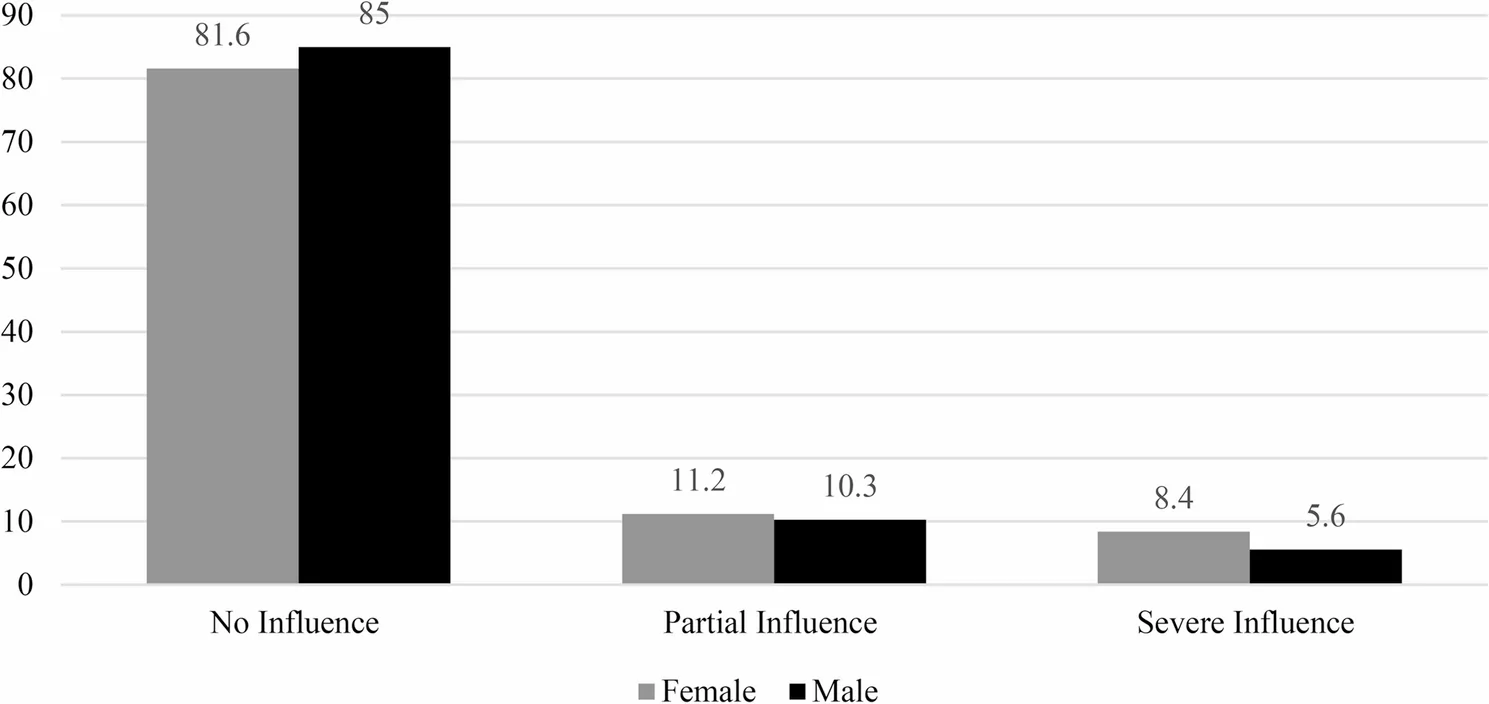
Self-reported impact of comorbidities on quality of life

Notably, 85.0% of males and 81.6% of females reported that their existing medical conditions exerted no perceived impact on their HRQoL (Fig. 2). However, statistical analysis showed a negative correlation between the w-FCI total score and the EQ-5D-3L index score, Spearman rho (ρ) = − 0.33, *p* < .001.

#### Weighted functional comorbidity index

The total median w-FCI score for the sample (*n* = 396) was 4, with median scores of 4 for females and 3 for males. Visual impairment was the most prevalent comorbidity, reported by 59.0% of females and 73.0% of males. Among females, the next most common conditions were obesity (37.1%), diabetes mellitus (34.0%), and arthritis (33.6%), whereas males most frequently reported arthritis (47.9%), obesity (32.1%), and degenerative disc disease (30.7%, Table 3). Multimorbidity was prevalent in 76.77% (females *n* = 196, males *n* = 108) of the participants.

**Table 3:** Weighted Functional Comorbidity Index (w-FCI) self-reported prevalence of comorbidities

Medical condition	Urban		Rural	
	Females (%)	Males (%)	Females (%)	Males (%)
Arthritis	71 (33.59)	49 (49.49)	15 (32.61)	18 (43.90)
Osteoporosis	49 (23.33)	17 (17.17)	10 (21.74)	9 (21.95)
Degenerative disc disease	51 (24.29)	28 (28.28)	13 (28.26)	15 (36.59)
Pulmonary disease	38 (18.10)	25 (25.25)	10 (21.74)	4 (9.76)
Angina pectoris	15 (7.14)	11 (11.11)	5 (10.87)	4 (9.76)
Myocardial infarction	11 (5.24)	4 (4.04)	5 (10.86)	0
Heart failure	9 (4.29)	5 (5.05)	4 (8.70)	1 (2.44)
Neurological disease	12 (5.71)	3 (3.03)	0	2 (4.88)
Dementia	0	0	0	0
Cerebrovascular accident	18 (8.57)	1 (1.01)	2 (4.35)	0
Peripheral vascular dx	22 (10.48)	9 (9.09)	4 (8.70)	8 (19.51)
Diabetes	69 (32.86)	33 (33.33)	18 (39.13)	9 (21.95)
Gastrointestinal disease	28 (13.33)	17 (17.17)	8 (17.39)	5 (12.20)
Obesity	79 (37.62)	31 (31.31)	16 (34.78)	14 (34.15)
Depression	47 (22.38)	24 (24.24)	10 (21.74)	11 (26.83)
Anxiety	46 (21.90)	21 (21.21)	10 (19.57)	10 (24.39)
Visual impairment	120 (57.14)	74 (74.75)	31 (67.39)	29 (70.73)

Total Sample *n* = 396, Urban Female *n* = 210, Rural Female *n* = 46, Urban Male *n* = 99, Rural Male *n* = 41

**Fig. 3:**
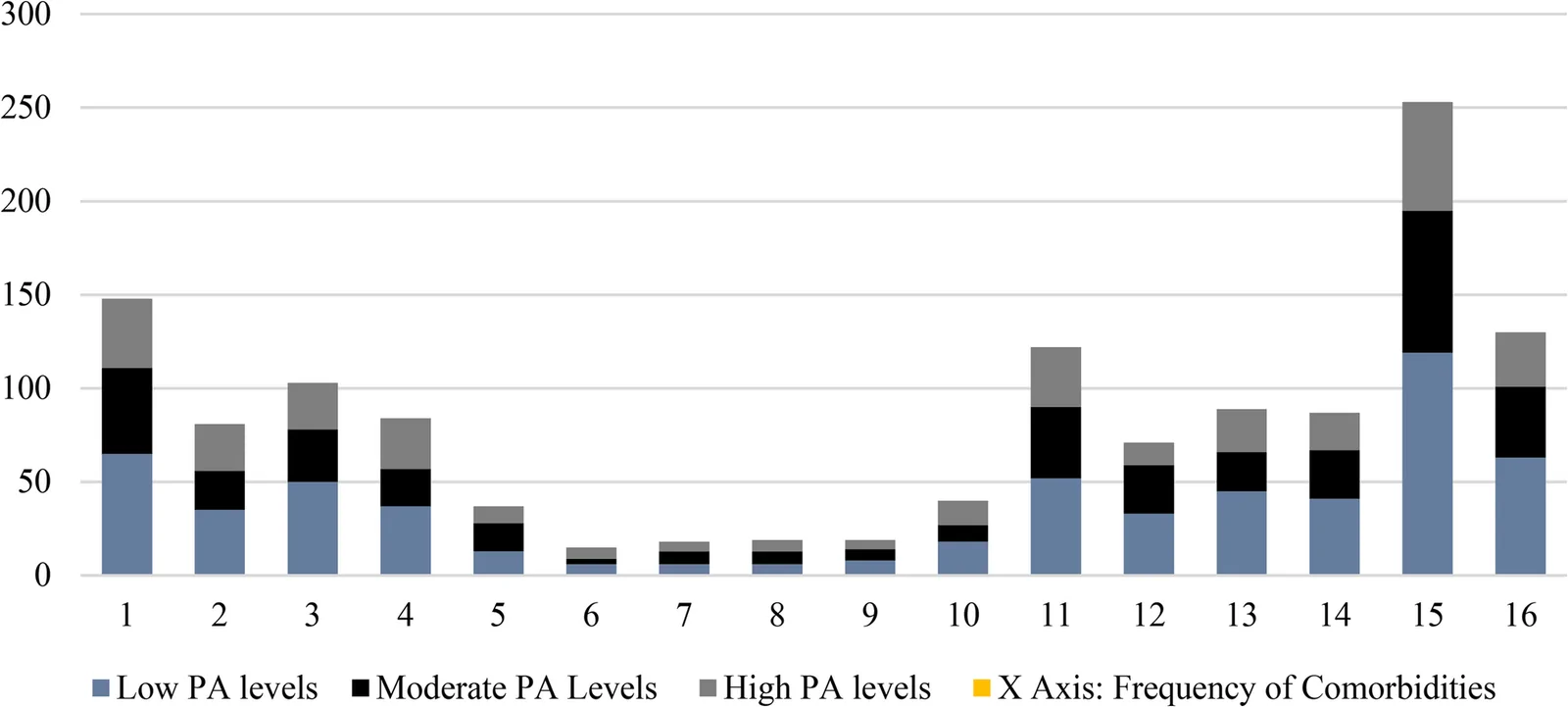
Frequency of comorbidities by physical activity levels. KEY 1 = Arthritis, 2 = Osteoporosis, 3 = DegenerativeDiscDx, 4 = PulmonaryDx, 5 = Angina Pectoris, 6 = Myocardial Infarction, 7 = Heart Failure, 8 = Neurological Dx,9 = CVA, 10 = Peripheral Vascular Dx, 11 = Diabetes, 12 = Gastrointestinal Dx, 13 = Depression, 14 = Anxiety, 15 = Visual Impairment, 16 = Obesity

Low, moderate, and high physical activity were reported in 189, 140, and 67 participants respectively. Participants who recorded low levels of physical activity demonstrated a higher frequency of arthritis, diabetes, degenerative disc disease, and obesity (Fig. 3).

TwoStep cluster analysis identified four distinct groups of adults in LTCF, each defined by certain combinations of chronic diseases. Table 4 summarises the size of the group and which health conditions are most common within them. The percentage shown for each cluster indicates how many adults from the entire study belong to that group. The percentages listed next to each disease show how common that specific condition is among adults within that cluster, rather than in the whole study. This helps us understand which diseases often appear together in different groups of older adults residing in LTCF. Participants exhibiting elevated waist-to-hip ratios demonstrated the highest cumulative frequency of comorbidities (*n* = 852), followed sequentially by those with low total scores on the Short Physical Performance Battery (*n* = 617) and by individuals reporting low levels of physical activity (*n* = 597).

**Table 4:** Multimorbidity Cluster Profiles

Cluster	Cluster Size (%)	Key Conditions (Prevalence in Cluster)
Cluster 1: Musculoskeletal and Vascular	32.1	Arthritis (47.1%), Osteoporosis (49.4%), Degenerative disc disease (48.6%), Peripheral vascular disease (51.2%), Angina (42.9%), Visual impairment (39.4%)
Cluster 2: Cardiovascular and Respiratory	33.8	Pulmonary disease (32.5%), Stroke/CVA (52.4%), Visual impairment (26.0%)
Cluster 3: Cardiometabolic and Neurological	10.1	Myocardial infarction (65.0%), Heart failure (57.9%), Degenerative disc disease (29.9%), Diabetes (17.8%), Neurological diagnoses (23.5%)
Cluster 4: Mental Health	24.0	Depression (85.9%), Anxiety (88.4%), Peripheral vascular disease (37.2%), Visual impairment (22.4%)

*CVA* Cerebrovascular Accident

Logistic binomial regression analysis was employed to predict the odds ratio for the presence of multimorbidity. Multimorbidity was held as a dichotomous constant against predictor variables of biological sex, age, residential status, marital status, educational level, heart rate, systolic blood pressure, diastolic blood pressure, oxygen saturation, BMI, hip circumference, waist circumference, waist-to-hip ratio, handgrip strength, SPPB (balance, gait speed, sit-to-stand, total), vigorous MET, moderate MET, walking MET, sedentary minutes, total MET, Barriers to Being Active Questionnaire scores, and EQ-5D-3L (mobility, self-care, usual activities, pain, anxiety, VAS, index score, total score) using enter and forward selection. In binomial logistic regression, the odds ratio, Exp(B), quantifies the association between each predictor and the binary outcome. It represents the change in odds of the outcome occurring with a one-unit increase in the predictor, while controlling for all other variables. The model predicting multimorbidity was statistically significant, χ²(140, *N* = 396) = 228.19, *p* < .001, with a specificity of 70.65%, sensitivity of 95.39%, and an overall model success rate of 89.65%. The significant predictors in the model for multimorbidity were:

- Age: Odds-ratio 1.13 - For each additional year of age, the odds of participants being classified with multimorbidity increased by approximately 13%.
- The EQ-5D-3L (depression): Odds-ratio 36.67 - Compared to those reporting no anxiety or depression, participants reporting moderate anxiety or depression were approximately 36.7 times more likely to be classified with multimorbidity.
- The EQ-5D-3L (pain): Odds-ratio 15.24 - Compared to those reporting no pain or discomfort, participants reporting pain or discomfort were approximately 15.2 times more likely to be classified with multimorbidity.
- The EQ-5D-3L (mobility): Odds-ratio 11.81 - Compared to those reporting no mobility problems, participants reporting difficulty walking were approximately 11.8 times more likely to be classified with multimorbidity.

#### Geriatric depression scale

Participants residing in urban settings exhibited a greater proportion with none-to-normal depression scores (67.64%) compared to their rural counterparts (55.20%, Table 5). A Spearman’s rank-order correlation was conducted to examine the relationship between GDS scores, EQ-5D-3L index and EQ-5D-3L VAS scores. The analysis revealed a significant negative correlation, ρ(394) = − 0.16, *p* < .01, and ρ(394) = − 0.28, *p* < .01, respectively.

**Table 5:** Depression classification of participants

Geriatric Depression Scale	Urban		Rural	
	Females (%)	Males (%)	Females (%)	Males (%)
Nil/normal	145 (69.04)	64 (64.65)	26 (56.52)	22 (53.66)
Mild	48 (22.86)	25 (25.25)	15 (32.62)	17 (41.46)
Moderate	15 (7.14)	9 (9.09)	5 (10.86)	1 (2.44)
Severe	2 (0.96)	1 (1.01)	0	1 (2.44)

Total Sample *n* = 396, Urban Female *n* = 210, Rural Female *n* = 46, Urban Male *n* = 99, Rural Male *n* = 41

Analysis of the depression screening tools correlation matrix reveals several notable patterns reflecting the degree of overlap and potential complementarity among instruments (Table 6).

**Table 6:** Depression screening tools matrix using Spearman’s correlation

	ICOPE screening Feeling down/ depressed	ICOPE screening Little interest in activities	Geriatric Depression Scale	EQ-5D-3 L depression	w-FCI – depression
ICOPE screening Felling down/depressed	-	0.66**	0.14**	0.37**	0.42**
ICOPE screening Little interest in activities	0.66**	**-**	0.22**	0.42**	0.51**
Geriatric Depression Scale	0.14**	0.22**	**-**	0.22**	0.23**
EQ-5D-3L depression	0.37**	0.42**	0.22**	**-**	0.62**
w-FCI – depression	0.42**	0.51**	0.23**	0.62**	**-**

*EQ-5D-3L* European Quality of Life 5-Dimensions 3-Level Version, *ICOPE* Integrated care of older adults, *w-FCI* Weighted Functional Comorbidity Index

**Correlation is significant at the 0.01 level (2-tailed)

Moderate correlations were observed between both ICOPE depression components and the EQ-5D-3L depression dimension (*ρ* = 0.37 and *ρ* = 0.42, *p* < .01) as well as with the w-FCI depression item (*ρ* = 0.42 and *ρ* = 0.51, *p* < .01). These findings suggest that while the ICOPE tool captures depressive symptoms effectively, its items reflect both emotional and functional aspects of depression, aligning well with broader health-related quality of life measures (EQ-5D-3L) and comorbidity assessments (w-FCI).

The EQ-5D-3L Visual Analogue Scale (VAS) emerged as a significant predictor of GDS scores (*β* = −0.02, SE = 0.007, Wald χ² = 12.05, *p* < .001) [44], with the negative coefficient indicating that higher self-rated health status is associated with lower levels of depressive symptoms. This finding underscores the strong inverse relationship between perceived overall health and depressive symptomatology in older adults, highlighting the utility of the EQ-5D-3L VAS as a sensitive indicator in mental health assessments within this population.

## Discussion

### ICOPE screening

Depression prevalence in our sample, based on results from the ICOPE screening tool, was 19.04%, with rural-residing women exhibiting the highest prevalence at 26.10%. A systematic review of studies using the ICOPE tool reported a combined prevalence of 17.90%, closely aligning with our findings [45]. The ICOPE depression screening tool demonstrated strong validity in detecting depression within this cohort, evidenced by its direct correlation with GDS, and EQ-5D-3L (depression), Spearman rho *ρ* = 0.22, *p* = .01. Its negative correlation with handgrip strength, consistent with similar associations observed for GDS and EQ-5D-3L, further validates its applicability for older adults in South Africa as handgrip strength has also been linked to depression in rural-residing populations [46].

### European quality of life 5 dimensions 3 level version

Our findings revealed that pain and mobility challenges were significant determinants negatively affecting participants’ HRQoL. Similarly, a study conducted among residents of an OAH in Bloemfontein, SA identified these domains as the primary factors influencing HRQoL, with EQ-5D-3L VAS scores aligning closely with our results [47]. A comprehensive multi-country study aggregating results from the EQ-5D-3L assessment identified mobility limitations and pain/discomfort as the predominant factors negatively affecting HRQoL in older adults, corroborating our findings [48]. However, the VAS scores reported for the countries included in the review were higher than those observed in our sample. Additionally, our study revealed a significant correlation between Short Physical Performance Battery scores and HRQoL, aligning with findings from a study on older adults following hospital discharge, which reported similar associations [49].

The significant predictors observed in our results between multimorbidity and key dimensions of HRQoL, specifically depression, pain, and mobility limitations, highlight the pervasive impact of multiple chronic conditions on older adults’ physical and psychological well-being. A systematic review exploring the relationship between multimorbidity and quality of life in older adults lends strong support to our findings, affirming that the cumulative burden of multiple chronic conditions can profoundly disrupt all aspects of daily living and well-being [50]. As displayed in our results older adults with multimorbidity were substantially more likely to report depressive symptoms, pain, and mobility challenges, indicating that the burden of co-existing diseases extends beyond clinical diagnoses to deeply affect daily functioning and quality of life. The review conducted by Nakad et al. (2020) aligns with our findings, providing evidence that pain and multimorbidity are intricately interrelated among older adults, with the presence of multiple chronic conditions significantly amplifying the burden of pain and its consequent impact on overall well-being [51]. The study by Chang et al. (2019) strengthens our findings, revealing that multimorbidity is associated with notable declines in both physical functioning and overall well-being among older adults, thereby highlighting the extensive impact of multiple chronic conditions on quality of life [14]. These findings reinforce the concept that multimorbidity is not merely a medical phenomenon, but a multidimensional challenge, intricately linked with emotional distress and physical disability. Addressing multimorbidity in this population therefore demands integrated care models that encompass both physical and mental health, aiming to mitigate its deleterious effects on HRQoL and to promote holistic well-being in LCTF.

### Multimorbidity

The high prevalence of multimorbidity observed in our study (76.77%) is higher than prior research conducted among community residing older adults in South Africa [7, 14, 20]. In our study, females bore a higher burden of multimorbidity, corroborating findings from research conducted among community-dwelling older adults’ and highlighting gender disparities in the prevalence and impact of multiple chronic conditions [14]. However, to date, no studies have specifically focused on populations residing within LTCF in Sub-Saharan Africa, underscoring a critical gap in the literature concerning this particularly vulnerable cohort. However, a global systematic review conducted by van den Brink et al. (2013), focusing predominantly on high-income countries, reported multimorbidity prevalence rates as high as 64.7% among older adults residing in LTCF [52]. Our results indicate that advancing age significantly increases the risk of multimorbidity, aligning with prior research conducted in cohorts of community-residing older individuals [7, 20].

The greatest burden of comorbidities in our study was observed among participants with high waist-to-hip ratios, followed by those with low SPPB scores and, thirdly, by individuals with low physical activity levels, highlighting key factors associated with multimorbidity in this cohort. A study conducted in India among community-dwelling older adults aligned with our findings, demonstrating a significant correlation between multimorbidity and elevated waist-to-hip ratios [53]. Although data are lacking for older adults in LCTF in Sub-Saharan Africa, highlighting a gap in research, a systematic review among community-dwelling older individuals confirmed a significant association between obesity and multimorbidity [20]. Consistent with our findings, Grund et al. (2017) reported an inverse relationship between multimorbidity and SPPB scores, emphasising that reduced physical performance predicts heightened risks of disability, institutionalisation, and adverse outcomes [54]. Vancampfort et al. (2017) conducted a comprehensive review across 46 lower-middle-income countries, revealing an inverse relationship between physical activity and multimorbidity in older adults, consistent with our study’s results [55].

The results of this study identified four distinct multimorbidity clusters among older adults in South African LTCF, revealing significant variation in disease patterns and highlighting a particularly high-burden subgroup with complex health needs. Cluster 2 accounted for 33.8% of participants and was defined by high rates of stroke (52.4%), pulmonary disease (32.5%), and visual impairment (26.0%). The coexistence of cardiovascular and respiratory conditions in this cluster signals a considerable health burden, suggesting a need for integrated care approaches to manage these interrelated diseases and reduce risks of further functional decline and hospitalisation [56]. Despite its smaller size (10.1%), Cluster 3 demonstrates a high burden of multimorbidity, marked by the coexistence of multiple severe chronic conditions, identifying it as a subgroup at significant risk for adverse health outcomes [20].

The inverse correlation between w-FCI scores and EQ-5D-3L index values indicates that greater multimorbidity burden objectively diminishes HRQoL, despite many participants perceiving no impact from their medical conditions. This discordance highlights the need for comprehensive assessments that integrate both subjective and objective health measures.

### Depression

We observed that older adults residing in rural LTCF, particularly women, exhibited a significantly higher prevalence of depressive symptoms compared to their urban counterparts. These findings align with a systematic review conducted by Purtle et al. (2019), who reported similar patterns among older populations in developing nations, thereby underscoring the heightened vulnerability of rural-dwelling individuals to mental health disparities within institutional contexts [57]. Cluster 4 includes nearly a quarter of the participants and is dominated by mental health issues, particularly depression and anxiety. These individuals also have higher rates of circulation problems and vision impairment, indicating complex health needs. This highlights the importance of providing mental health care alongside physical health services in LTCF. Higher GDS scores were significantly negatively correlated with both EQ-5D-3L index values and VAS scores, indicating that greater depressive symptoms were linked to poorer HRQoL and lower self-rated overall health. Peltzer et al. (2013) also observed that higher levels of depression were linked to poorer quality of life among older adults, aligning closely with our current results [58]. The review conducted by Siversten et al. (2015) confirmed a persistent association between greater depression severity and poorer quality of life among older adults, consistent across time and irrespective of the assessment tools employed [59].

Higher GDS scores were significantly correlated with a greater number of comorbidities, *ρ* = 0.28, *p* < .01, demonstrating the potential impact of depression on the burden of multimorbidity in older adults. Similar associations have been reported in studies involving older adults residing in community settings, however, to date, no research has examined these relationships among older populations in LTCF within sub-Saharan Africa [7, 20].

The high prevalence of multimorbidity, coupled with its strong association with lower HRQoL, presents significant challenges for older adults as they age within LTCF. As individuals grow older, the cumulative burden of multiple chronic conditions accelerates functional decline, increases vulnerability to physical and psychological distress, and complicates care requirements, thereby profoundly impacting their capacity to maintain independence and overall well-being in institutional settings. The Geriatric Depression Scale exhibited relatively weaker, yet still statistically significant, correlations with other instruments screening for depression in the study (Table 6). This modest association suggests that while the GDS captures depressive symptoms, it may assess more nuanced or specific psychological constructs than brief screening tools like EQ-5D-3L, potentially accounting for its lower magnitude correlations. Of particular note, the strongest association in the matrix was observed between the EQ-5D-3L depression dimension and the w-FCI depression item (*ρ* = 0.62, *p* < .01), highlighting substantial shared variance. This strong link suggests that depressive symptoms substantially influence perceived health-related quality of life and may co-occur with comorbidities that affect functional status. Collectively, these findings demonstrate both convergence and divergence among the instruments. While all tools capture aspects of depressive symptomatology, their differing correlation strengths imply that each may emphasise distinct dimensions, ranging from emotional symptoms (ICOPE, GDS) to functional impact (w-FCI, EQ-5D-3L). This has significant implications for tool selection in clinical practice and research, suggesting that using multiple measures may offer a more comprehensive assessment of depression in older adults, particularly in settings like LTCF where functional and psychological health are closely intertwined.

## Conclusion

This study underscores the stark reality of “Aging with Burden” among older adults residing in LTCF, revealing a strikingly high prevalence of multimorbidity and its profound ramifications for HRQoL. The delineation of four distinct multimorbidity clusters highlights that aging is far from a homogeneous process. It is rather characterised by diverse and complex health trajectories, particularly among individuals contending with cardiometabolic and mental health conditions. Moreover, the pronounced associations between depressive symptoms, multimorbidity, and diminished HRQoL illuminate the intricate nexus between somatic and psychological health in shaping the lived experiences of older adults.

Importantly, this study also demonstrates that multimorbidity frequently coexists with poor physical function, central adiposity, and pervasive physical inactivity, synergistic factors that further erode functional capacity, amplify disability risk, and exacerbate the decline in quality of life. Collectively, these findings make clear that aging in this context is accompanied by significant medical, functional, and psychological burdens, necessitating integrated, person-centred interventions that holistically address the multidimensional challenges posed by multimorbidity. Addressing this multifaceted burden is imperative, not only to enhance the health, autonomy, and quality of life of institutionalised older adults across sub-Saharan Africa, but also to inform and advance global geriatric practice toward more equitable, effective, and compassionate care. This is particularly crucial for those residing in rural settings, and even more so for older women, who remain disproportionately vulnerable to the compounded impacts of multimorbidity and psychosocial adversity.

## Limitations

The cross-sectional nature of this study inherently constrains the ability to draw causal inferences regarding the observed associations. Furthermore, the deliberate selection of relatively healthy older adults, necessitated by stringent eligibility criteria, may limit the extrapolation of findings to more frail or clinically complex populations. The study’s geographic confinement to a single South African province underscores the need for broader, multicentric investigations to adequately capture regional heterogeneity and sociocultural variability. Moreover, the paucity of comparable data from LTCFs across sub-Saharan Africa impeded nuanced contextualisation, compelling reliance on community-dwelling cohorts and global LTCF literature to interpret and situate the findings. These limitations collectively highlight the imperative for future research to expand geographic scope, encompass more diverse clinical profiles, and generate region-specific evidence to inform tailored interventions for institutionalised older adults in this setting. Multimorbidity was operationalised using the w-FCI, a function-oriented index that includes obesity due to its impact on physical performance. As obesity is frequently considered a risk factor rather than a disease, its inclusion may have influenced multimorbidity prevalence estimates. However, multimorbidity classification required at least two comorbid conditions, and obesity alone did not define disease burden. This should be considered when interpreting prevalence estimates and comparing findings with studies using disease-count approaches.

## Supplementary Information

Supplementary Material 1.

## Data Availability

The data that support the findings of this study are not publicly available due to ethical restrictions imposed by the University of Cape Town Human Research Ethics Committee (HREC/325/2023) to protect participant confidentiality. Data access requests may be directed to the University of Cape Town HREC or the corresponding author and will be considered subject to appropriate ethical approval.
