# Supplementary material for: Aging with burden: multimorbidity, depression and quality of life in older adults residing in long-term care facilities in South Africa
Source: BMC Geriatr. 2026 Jun 27;26:1172. doi: 10.1186/s12877-026-07846-4 (PMC13584380; doi:10.1186/s12877-026-07846-4)
Supplement: Supplementary file 1 [file 12877_2026_7846_MOESM1_ESM.docx]

Supplementary File 1: Screening Outcomes

|  | | Urban | | Rural | | Total |
| --- | --- | --- | --- | --- | --- | --- |
|  |  | **Female** | **Male** | **Female** | **Male** |  |
| Age (years) | Mean | 72.54 | 71.65 | 77.24 | 72.59 | 72.88 |
| Biological sex |  | 210 | 99 | 46 | 41 | 396 |
| Marital status | Single | 34 | 25 | 12 | 19 | 90 |
|  | Married | 39 | 30 | 10 | 7 | 86 |
|  | Divorced | 99 | 19 | 20 | 10 | 148 |
|  | Widowed | 38 | 25 | 4 | 5 | 72 |
| Education | No formal education | 7 | 1 | 5 | 4 | 17 |
|  | Primary school | 38 | 7 | 15 | 10 | 70 |
|  | Secondary school | 146 | 76 | 25 | 25 | 272 |
|  | Diploma | 14 | 10 | 1 | 0 | 25 |
|  | Degree | 5 | 5 | 0 | 2 | 12 |
| ICOPE Cognition | Orientation to date and place | 210 | 99 | 46 | 41 | 396 |
|  | Recalls three easy words correctly | 85 | 42 | 14 | 19 | 160 |
| ICOPE Mobility | 5 successful unassisted chair stands in 14 seconds | 179 | 86 | 37 | 32 | 334 |
| ICOPE Malnutrition | Have you unintentionally lost > 3kg in the last 3 months. | 10 | 2 | 2 | 1 | 15 |
|  | Have you lost your appetite? | 28 | 5 | 6 | 4 | 43 |
| ICOPE Vision | Do you have problems with your eyes, difficulties in seeing far, reading, eye disease or currently under medical treatment (e.g. Diabetes or high BP) | 146 | 56 | 25 | 22 | 249 |
| ICOPE Hearing | Pass whisper test | 210 | 99 | 46 | 41 | 396 |
| ICOPE Depression | Over the last 2 weeks have you been bothered by – feeling down or depressed. | 36 | 14 | 11 | 6 | 67 |
|  | Little interest in doing things. | 47 | 22 | 13 | 4 | 86 |
| Resting heart rate (bpm) | Mean | 78.89 | 78.07 | 81.04 | 78.41 | 78.89 |
| SBP (mmHg) | Mean | 130.48 | 127.71 | 132.61 | 126.32 | 129.60 |
| DBP (mmHg) | Mean | 76.24 | 77.05 | 80.98 | 77.80 | 77.16 |
| Oxygen saturation (SpO_2_) | Mean | 98.72 | 98.89 | 98.68 | 98.77 | 97.92 |
| BMI (kg/m^2^) | Mean | 27.88 | 26.72 | 27.77 | 27.40 | 27.53 |
| Weight status (*n*) | Underweight | 6 | 1 | 2 | 1 | 10 |
|  | Normal | 65 | 42 | 16 | 11 | 134 |
|  | Overweight | 64 | 27 | 16 | 19 | 126 |
|  | Obese | 75 | 29 | 12 | 10 | 126 |
| GDS total score | Median | 3 | 3 | 4 | 4 | 3 |
| GDS depression scale (*n*) | Nil | 145 | 64 | 26 | 22 | 257 |
|  | Mild | 48 | 25 | 15 | 17 | 105 |
|  | Moderate | 15 | 9 | 5 | 1 | 30 |
|  | Severe | 2 | 1 | 0 | 1 | 4 |
| Waist-hip ratio | Mean | .91 | .99 | .91 | .94 | .93 |
| MET min/week (mean) | Vigorous | 209.33 | 124.65 | 126.09 | 275.12 | 185.30 |
|  | Moderate | 476.19 | 422.06 | 292.61 | 222.93 | 415.11 |
|  | Walking | 1094.58 | 1214.67 | 1007.93 | 991.61 | 1103.86 |
|  | Total | 1693.95 | 1674.73 | 1401.72 | 1267.22 | 1611.02 |
| Sedentary minutes | Mean | 151.64 | 153.54 | 150.43 | 157.56 | 152.59 |
| HGS (kg) | Mean | 14.46 | 19.90 | 12.85 | 24.19 | 16.64 |
| SPPB total | Median | 7 | 7 | 6 | 7 |  |
| EQ-5D-3L VAS | Mean | 70.30 | 71.52 | 69.78 | 69.51 | 70.46 |
| EQ-5D-3L index | Mean | .82 | .85 | .83 | .86 | .83 |
| w-FCI score | Median | 4 | 3 | 4 | 3 | 4 |
| Number of comorbidities | Median | 3 | 3 | 3 | 2 | 3 |

Total Sample *n* = 396, Urban Female *n* = 210, Rural Female *n* = 46, Urban Male *n* = 99, Rural Male *n* = 41. ICOPE: Integrated Care for Older People, SBP: Systolic blood pressure, DBP: Diastolic blood pressure, BMI: Body mass index, GDS: Geriatric Depression Scale, HGS: Handgrip strength, SPPB: Short Performance Physical Battery, EQ-5D-3L VAS: The European Quality of Life 5-Dimension 3-Level Visual Analogue Scale, w-FCI: Weighted Functional Comorbidity Index
